# Administration of glycerol-based formulations in sheep results in similar ovulation rate to eCG but red blood cell indices may be affected

**DOI:** 10.1186/s12917-020-02418-z

**Published:** 2020-06-22

**Authors:** Cristian Porcu, Francesca D. Sotgiu, Valeria Pasciu, Maria Grazia Cappai, Alicia Barbero-Fernández, Antonio Gonzalez-Bulnes, Maria Dattena, Marilia Gallus, Giovanni Molle, Fiammetta Berlinguer

**Affiliations:** 1 Department of Veterinary Medicine, University of Sassari, Via Vienna 2, 07100 Sassari, Italy; 2 Universidad Alfonso X el Sabio, Campus de Villanueva de la Cañada, Avd. Universidad 1, 28040 Madrid, Spain; 3 Comparative Physiology Group, SGIT-INIA, Av. Puerta de Hierro s/n, 28040 Madrid, Spain; 4 AGRIS Sardegna, Loc. Bonassai, 07100 Sassari, Italy

**Keywords:** Propylene glycol, Ovary, Insulin, Glucose, NEFA, Urea, Plasma osmolality, Red blood cells

## Abstract

**Background:**

The objective of this study was to investigate the metabolic and osmotic effects of different doses of glycerol or a glycerol – propylene glycol mixture in Sarda sheep with the aim to identify those able to beneficially modify ewe’s metabolic status without harmful changes in red blood cell (RBC) indices. Thereafter, the selected doses were tested for their effects on ewe’s ovarian activity during an induced follicular phase and compared to the effects of a hormonal treatment with equine chorionic gonadotrophin (eCG).

**Results:**

Glycerol was administered alone (G groups: 90% glycerol and 10% water; % v/v) or in combination with propylene glycol (M groups: 70% glycerol, 20% propylene glycol, 10% water; % v/v). Treatments were formulated to provide 100, 75, 50 and 25% of the amount of energy supplied in previous experiments. Obtained results showed that the formulations G75 and M75 (22.5 and 18.2% on DM basis, respectively) induce metabolic changes comparable to those induced by M100. The latter dose has been already evaluated for its effects on sheep metabolism and reproductive performance. However, with these high doses, plasma osmolality increased significantly, and RBC indices showed significant alterations. The low dose groups (G25 and M25, 8.6 and 6.9% on DM basis, respectively) did not show any alterations in plasma osmolality and RBC indices, but the metabolic milieu differed markedly from that of M100. Between the medium dose groups, M50 (12.9% on DM basis) showed a more comparable milieu to M100 than G50 (15.9% on DM basis) and no RBC alterations. Therefore, M75, G75 and M50 doses were tested for their effect on ovarian functions and proved to be equally effective as eCG.

**Conclusion:**

The results of the present study evidenced an alteration of RBC indices, and possibly of their functions, as a side effect of glycerol administration at high doses in the diet of ewes. Therefore, protocols foreseeing the administration of glycerol should be tested for their effects on RBC indices and functions. In general terms, the medium dose of the glucogenic mixture (12.9% of dietary DM on offer) should be preferred.

## Background

By-products sourced from plant crops represent an important human-inedible feed resource for livestock production [1]. These waste streams can source from different agro-industrial processes, and their use as feed ingredient or feed supplement can contribute to the environmental sustainability of livestock sectors. Recently, there has been an increasing global demand for biofuels, resulting in an increased demand for the feedstuffs (corn, wheat, and oilseeds) used for fuel extraction. The main by-product during the production of biodiesel is glycerol, which can be used as livestock feed ingredient or feed supplement [2]. The EU legislation approved glycerol and another biodiesel co-product, namely propylene glycol, as animal feed additives with no restrictions on animal species or quantity that may be fed, while recommending from the European Food Safety Authority the collection of data on the presence of impurities and contaminants in crude glycerine from biodiesel production [3, 4].

In small ruminants, glycerol, alone or in association with propylene glycol, has been used both as replacer of corn grain in the diet [5–9] and as a feed supplement to increase productive performance [10–17]. In these studies, glycerol was supplemented at a percentage of inclusion on dry matter (DM) basis ranging from 3% up to 45%. Overall the metabolic effects described include an increase in glucose and a decrease in NEFA circulating concentrations [6, 11, 18]. Other studies in ewes also reported an increase in insulin [19–21], insulin-like growth factor-1 [20], and a decrease in urea circulating concentrations [20, 21] after oral administration of 280 mL glycerol and 80 mL 1,2- propylene glycol daily for 4 days. These changes have been associated with changes in follicular fluid composition during treatment period [20], and may contribute in creating a suitable systemic and local metabolic milieu for the promotion of ovarian function. Glucogenic mixtures based on glycerol, administered as short-term flushing can indeed increase ovulation rate [19] and oocyte quality [10] in sheep. The increase in ovulation rate following glycerol administration has been described also in other studies in which glycerol was administered in a single dose [14, 15]. Therefore, a short or very short (daily) flushing based on glycerol can represent a valid alternative to other short flushing techniques based on conventional feedstuff such lupin seeds [22] or soybean meal [23, 24]. Moreover, thanks to its boosting activity on ovarian functions, glycerol could possibly substitute for synthetic gonadotropins dosed to increase lambing and twinning rates in sheep. Among them, it is noteworthy the equine chorionic gonadotropin (eCG), a hormone commonly used in concert with progestogen to induce ovulation prior to natural mating or artificial insemination. The future use and availability of eCG appears to be strongly challenged by highly active animal-rights movement because the hormone is obtained from pregnant mares. Hence, there is a need for alternative synchronization protocols without eCG [25].

Although there is evidence that glycerol and glucogenic mixtures can improve production and reproductive performance in sheep, these additives may sometimes negatively impact on animal welfare, at least under some circumstances. In fact, after oral administration, a significant proportion of glycerol (44% according to [26]) is rapidly absorbed through rumen wall by passive diffusion and transported via the bloodstream to serve directly as a substrate for glucose synthesis in the liver [27, 28]. In a previous study [21], we reported that the administration of a glycerol-based formulation (280 mL glycerol and 80 mL propylene glycol for 4 days; 23% DM) caused a 400-folds increase in circulating glycerol concentration in dairy ewes. Unfortunately, glycerol is a hyperosmotic agent and, consequently, plasma osmolality was greatly increased [21]. This effect had been already observed in humans [29–31]. Glycerol can readily permeate the red blood cells (RBCs) membrane at body temperature following a concentration gradient [32]. Both the increase in plasma osmolality [33] and glycerol diffusion in the RBCs [34] can impact on cellular volume homeostasis which is critical for erythrocyte survival [35]. Changes in RBCs volume can affect their membrane integrity, leading to impaired functionality and increased eryptosis [36, 37]. The critical importance of RBC volume regulation is demonstrated by several pathologies resulting from either overhydration or dehydration of RBCs [33].

Thus, the objective of this study was to investigate the metabolic and osmotic effects of different doses of glycerol and glycerol – propylene glycol mixtures in Sarda sheep in order to identify those able to positively modify ewe’s metabolic status without significantly affecting RBC indices. The highest dose of the glucogenic mixture was taken from previous studies, being already evaluated for its effects on sheep metabolism and reproductive performance [8, 16, 18]. Thereafter, to determine the ability of the selected glucogenic doses to promote ovarian function, we evaluated their effects on ewe’s ovarian activity during an induced follicular phase, comparing them to the effects of the administration of a synthetic gonadotropin (eCG).

## Results

### Phase 1

Body weight did not change after the administration of the dietary treatments and no differences were observed between groups (M100 51.6 ± 1.7, G100 53.8 ± 3, M75 51.6 ± 2.1, G75 50.3 ± 1.8, M50 48.7 ± 1.9, G50 48.1 ± 1.8, M25 50.1 ± 1.8, G25 50.6 ± 3.8; *P* > 0.05).

Before starting the titration feeding treatments, plasma osmolality, RBC indices, circulating concentrations of analysed metabolites and hormones were within the physiological ranges for the species [38] in all the ewes showing no differences between groups (D 0; Table 1).

**Table 1:** Plasma osmolality, RBC indices and circulating concentrations of analysed metabolites and hormones

Group	Day	OSMOL/kg	MCV	MCH	MCHC		RDW-Sd	GLUCOSE	UREA		NEFA		INSULIN	GLYCEROL	
			(fL)	(pg)	(g/L)		(fL)	mg/dL	mg/dL		Mmol/L		μg/L	mg/dL	
M100	D0	0.317 ± 0.002	34.50 ± 0.78	11.24 ± 0.27	313.00 ± 2.93	^a^	28.48 ± 0.78	55.94 ± 3.52	21.65 ± 2.20	^a^	0.14 ± 0.01	^a^	0.28 ± 0.06	0.09 ± 0.03	
M100	D3	0.316 ± 0.001	34.36 ± 0.78	11.12 ± 0.23	324.40 ± 2.56		28.32 ± 0.84	63.96 ± 4.59	13.92 ± 2.10		0.06 ± 0.01		0.40 ± 0.07	0.05 ± 0.00	
G100	D0	0.317 ± 0.003	36.22 ± 0.91	11.40 ± 0.27	314.80 ± 2.65		30.60 ± 0.70	49.42 ± 1.25	24.10 ± 2.58	^a^	0.11 ± 0.05		0.50 ± 0.05	0.14 ± 0.04	
G100	D3	0.318 ± 0.005	36.02 ± 0.86	11.50 ± 0.29	319.60 ± 6.04		30.34 ± 0.61	55.23 ± 2.59	12.38 ± 1.45		0.05 ± 0.02		0.66 ± 0.11	0.13 ± 0.05	
M75	D0	0.275 ± 0.035	35.88 ± 1.32	11.30 ± 0.30	303.60 ± 5.00		31.22 ± 1.18	52.63 ± 3.73	25.58 ± 1.75	^a^	0.18 ± 0.08		0.52 ± 0.04	0.20 ± 0.08	
M75	D3	0.315 ± 0.003	37.24 ± 1.11	11.36 ± 0.29	304.80 ± 6.26		30.62 ± 1.20	59.30 ± 2.47	12.87 ± 0.80		0.02 ± 0.00		0.70 ± 0.19	0.19 ± 0.12	
G75	D0	0.308 ± 0.004	33.44 ± 1.11	10.48 ± 0.33	311.20 ± 2.78		28.06 ± 1.07	52.28 ± 1.36	22.07 ± 0.61	^a^	0.31 ± 0.05	^a^	0.56 ± 0.11	0.16 ± 0.04	
G75	D3	0.309 ± 0.003	33.44 ± 1.11	10.42 ± 0.34	311.40 ± 2.87		27.96 ± 1.01	53.23 ± 4.19	10.95 ± 1.38		0.04 ± 0.01		0.72 ± 0.18	0.17 ± 0.07	
M50	D0	0.300 ± 0.000	35.84 ± 1.32	11.44 ± 0.25	305.60 ± 6.65		31.44 ± 1.34	52.16 ± 2.21	26.80 ± 2.08	^a^	0.15 ± 0.05	^a^	0.32 ± 0.08	0.23 ± 0.07	^a^
M50	D3	0.302 ± 0.001	37.32 ± 1.09	11.44 ± 0.29	307.00 ± 5.43		31.42 ± 1.29	57.56 ± 1.13	17.06 ± 1.38		0.03 ± 0.01		0.40 ± 0.09	0.10 ± 0.03	
G50	D0	0.311 ± 0.007	33.58 ± 0.85	10.48 ± 0.32	312.00 ± 2.35		27.50 ± 0.99	42.43 ± 7.01	29.19 ± 2.02	^a^	0.12 ± 0.02		0.47 ± 0.12	0.38 ± 0.08	
G50	D3	0.304 ± 0.016	33.34 ± 0.80	10.36 ± 0.30	310.60 ± 2.82		27.76 ± 0.98	53.33 ± 2.36	18.32 ± 1.22		0.05 ± 0.03		0.50 ± 0.16	0.17 ± 0.06	
M25	D0	0.311 ± 0.003	34.66 ± 0.88	10.86 ± 0.22	313.00 ± 2.93		29.24 ± 1.01	55.94 ± 3.52	21.65 ± 2.20		0.23 ± 0.06		0.27 ± 0.06	0.12 ± 0.07	
M25	D3	0.317 ± 0.004	34.70 ± 0.84	11.02 ± 0.28	318.20 ± 2.52		29.34 ± 0.99	46.19 ± 3.52	16.54 ± 2.59		0.18 ± 0.03		0.33 ± 0.08	0.06 ± 0.02	
G25	D0	0.307 ± 0.001	36.50 ± 0.82	11.36 ± 0.20	311.80 ± 3.40		29.96 ± 1.01	48.26 ± 2.03	24.10 ± 2.58		0.25 ± 0.08	^a^	0.32 ± 0.11	0.10 ± 0.05	
G25	D3	0.306 ± 0.002	36.76 ± 0.87	11.36 ± 0.18	309.60 ± 3.61		30.50 ± 1.13	50.44 ± 2.74	20.28 ± 3.04		0.04 ± 0.01		0.33 ± 0.08	0.28 ± 0.18	

^a^Letters indicate significant differences between D 0 and D 3 (before glycerol-based formulation administration) within the same group; *p* < 0.05

To evaluate the osmotic effect of glycerol rise in the bloodstream, we assessed the changes in plasma osmolality and RBC indices among the different groups before and 2 h after glucogenic formulations administration. Pearson’s correlation analyses showed that the moles of glycerol and propylene glycol administered daily showed a moderately strong direct correlation (r = 0.668; *p* < 0.001) with plasma osmolality (Table 2).

**Table 2:** Pearson correlation coefficients between moles of glucogenic compounds administered, osmolarity, metabolites and hormones

	Moles of glycerol + propylene glycol	Plasma osmolality	Glycerol (mg/dL)	Glucose (mg/dL)	Insulin (μg/L)	UREA (mg/dL)
Plasma osmolality	0.668 (<0.001)					
Glycerol (mg/dL)	0.592 (<0.001)	0.830 (<0.001)				
Glucose (mg/dL)	0.352 (<0.001)	0.491 (<0.001)	0.493 (<0.001)			
Insulin (μg/L)	0.416 (<0.001)	0.308 (<0.001)	0.328 (<0.001)	0.425 (<0.001)		
UREA (mg/dL)	- 0.574 (<0.001)	- 0.357 (<0.001)	- 0.328 (<0.001)	- 0.300 (<0.001)	- 0.452 (<0.001)	
NEFA (mmol/L)	- 0.416 (<0.001)	- 0.304 (<0.001)	- 0.325 (<0.001)	- 0.226 (<0.001)	- 0.352 (<0.001)	0.175 (<0.01)

Corresponding *P*-values are shown within parenthesis

The administration of the glucogenic formulations determined indeed a significant increase in plasma osmolality in the high and moderate dose groups (M100, G100, M75, G75; p < 0.001). In these latter groups peak values were significantly higher than those recorded in the medium (M50 and G50) and low (M25 and G25;) dose groups (*p* < 0.05, Table 3).

**Table 3:** Plasma osmolality (Osm/kg) in consecutive blood samples drawn on D 3 of glucogenic mixture examinations

	00:00 h		00:30 h		01:00 h			01:30 h			02:00 h			03:00 h			04:00 h			10:00 h	
M100	0.316 ± 0.002	a	0.331 ± 0.003	ab	0.351 ± 0.007	AB	bc	0.370 ± 0.007	A	c	0.359 ± 0.005	A	bc	0.373 ± 0.009	A	c	0.365 ± 0.007	A	c	0.326 ± 0.004	ab
G100	0.318 ± 0.005	a	0.340 ± 0.006	ab	0.364 ± 0.007	A	b	0.369 ± 0.012	A	b	0.361 ± 0.003	A	b	0.373 ± 0.010	A	b	0.369 ± 0.008	A	b	0.315 ± 0.001	a
M75	0.315 ± 0.003	a	0.332 ± 0.002	ab	0.343 ± 0.003	ABC	ab	0.355 ± 0.006	ABC	b	0.356 ± 0.005	A	b	0.357 ± 0.007	A	b	0.346 ± 0.005	A	ab	0.316 ± 0.001	a
G75	0.309 ± 0.003	a	0.326 ± 0.002	ab	0.350 ± 0.009	AB	b	0.357 ± 0.008	AC	bc	0.363 ± 0.008	A	c	0.363 ± 0.006	AB	c	0.358 ± 0.003	A	bc	0.309 ± 0.001	a
M50	0.302 ± 0.001		0.313 ± 0.003		0.317 ± 0.003	BC		0.323 ± 0.004	BD		0.322 ± 0.006	B		0.320 ± 0.004	C		0.311 ± 0.001	B		0.305 ± 0.000	
G50	0.304 ± 0.006		0.316 ± 0.004		0.339 ± 0.009	ABC		0.331 ± 0.006	CD		0.324 ± 0.020	B		0.333 ± 0.006	BC		0.324 ± 0.006	B		0.309 ± 0.003	
M25	0.316 ± 0.004		0.316 ± 0.001		0.321 ± 0.004	BC		0.320 ± 0.005	D		0.316 ± 0.004	B		0.313 ± 0.004	C		0.311 ± 0.004	B		0.317 ± 0.004	
G25	0.306 ± 0.002		0.313 ± 0.003		0.311 ± 0.002	C		0.315 ± 0.006	D		0.316 ± 0.005	B		0.314 ± 0.001	C		0.311 ± 0.005	B		0.316 ± 0.003	

Different letters indicate significant differences (GLM UNIANOVA; p < 0.001): upper-case letters indicate differences between groups within the same time point; lower-case letters indicate differences within the same group at different time points

RBC indices and their variations after treatment are shown in Table 4. The absence of microscopic platelet clumping or RBC agglutination or roulaux formation in whole blood smears allowed to consider blood samples processed at automatic cell counter as diagnostic for the purposes of this investigation. It is to point out that the absence of roulaux bodies may also have a diagnostic indication for particular conditions, including erythrocyte sedimentation rate (ESR) which may occur in the presence of an inflammatory condition, for instance, despite it is consolidated that the two parameters may not be strictly proportional [39]. In view of the diagnostic aspects, all animals enrolled appeared clinically healthy throughout the trial.

**Table 4:** RBC indices in two consecutive blood samples drawn before and 2 h after glucogenic administration

	SAMPLE TIME	MCV (fL)		MCH (pg)	MCHC (g/L)		RDW-SD (fL)	
M100	00:00 h	34.36 ± 0.78	**	11.12 ± 0.23	324.40 ± 2.56	**	28.32 ± 0.84	*
	02:00 h	37.86 ± 0.58		11.20 ± 0.21	296.00 ± 5.74		32.24 ± 1.20	
G100	00:00 h	36.02 ± 0.86	*	11.50 ± 0.29	319.60 ± 6.04	*	30.34 ± 0.61	*
	02:00 h	41.74 ± 1.55		11.58 ± 0.28	278.80 ± 11.3		37.06 ± 2.00	
M75	00:00 h	37.24 ± 1.11	*	11.36 ± 0.29	304.80 ± 6.26	*	30.62 ± 1.20	*
	02:00 h	41.02 ± 1.10		11.46 ± 0.27	279.20 ± 7.29		35.48 ± 1.24	
G75	00:00 h	33.44 ± 1.11	*	10.42 ± 0.34	311.40 ± 2.87	***	27.96 ± 1.01	*
	02:00 h	38.22 ± 1.47		10.60 ± 0.37	277.60 ± 3.08		33.86 ± 1.62	
M50	00:00 h	37.32 ± 1.09		11.44 ± 0.29	307.00 ± 5.43		31.42 ± 1.29	
	02:00 h	38.70 ± 0.84		11.40 ± 0.22	294.40 ± 4.64		33.36 ± 1.18	
G50	00:00 h	33.34 ± 0.80		10.36 ± 0.30	310.60 ± 2.82	*	27.76 ± 0.98	
	02:00 h	35.70 ± 1.37		10.42 ± 0.32	292.00 ± 3.56		29.38 ± 0.32	
M25	00:00 h	34.70 ± 0.84		11.02 ± 0.28	318.20 ± 2.52		29.34 ± 0.99	
	02:00 h	34.98 ± 0.80		10.90 ± 0.22	311.60 ± 1.78		29.22 ± 0.93	
G25	00:00 h	36.76 ± 0.87		11.36 ± 0.18	309.60 ± 3.61		30.50 ± 1.13	
	02:00 h	37.00 ± 0.51		11.42 ± 0.32	308.80 ± 3.53		30.50 ± 0.55	

00:00 h: immediately before the morning administration of glucogenic mixture. MCV: defines the size of the red blood cells and is expressed as femtoliters (10^−15^; fl); MCH: quantifies the amount of haemoglobin per red blood cell and is expressed in picograms (pg); MCHC: indicates the amount of haemoglobin per unit volume and it is expressed as g/L of RBCs; RDW-SD: represents the coefficient of variation of the RBC volume distribution (size) and is expressed as femtoliters

* Asterisks indicate significant differences within the same group between time 00.00 and time 02.00: * p < 0.05; ** *p* < 0.01; *** p < 0.001

**Fig. 1:**
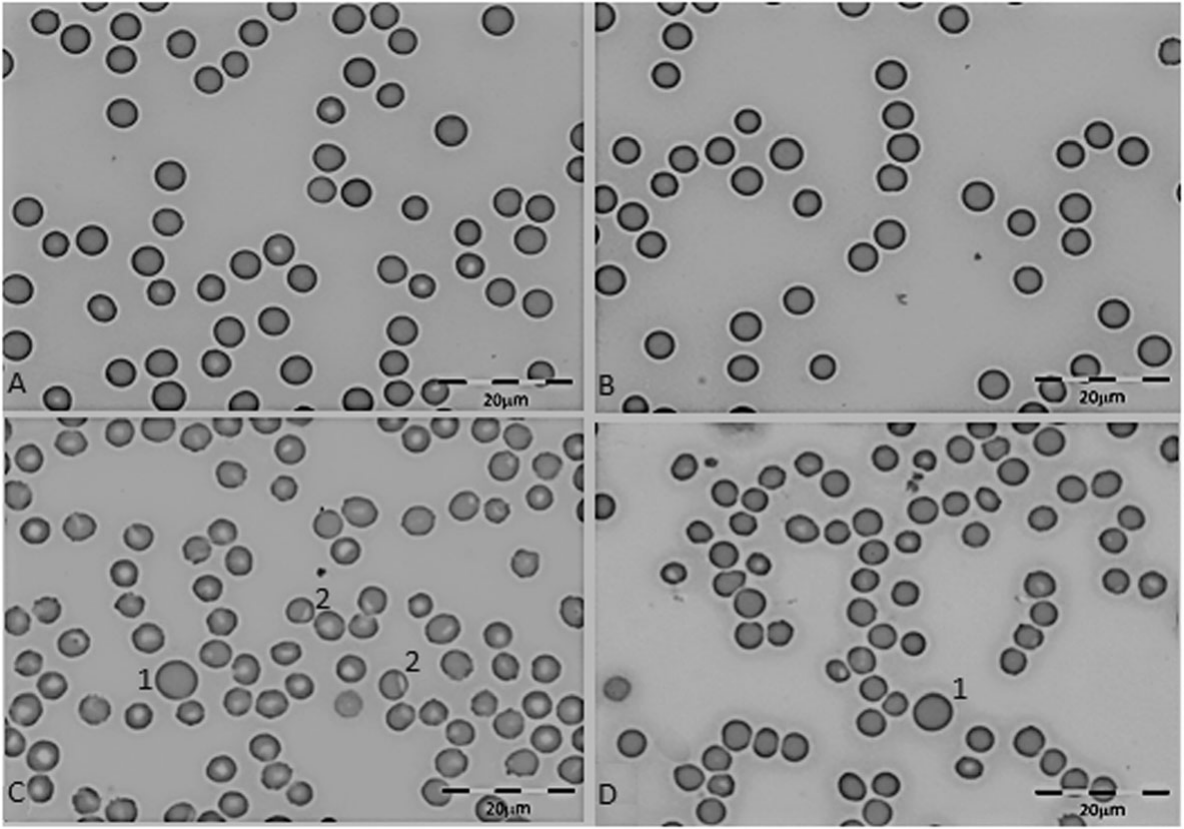
Representative images of RBC shape alterations observed in the high (M100 and G 100) and moderate dose (M75 and G75) groups. Panel **a** and **b** shows normal erythrocytes. Panel **c** show erythrocytes anisocytosis, macrocytosis (C-1), eccentrocyte (C-2) (hemoglobin halo is moved on one side showing oxidative damage). Panel D, erythrocytes anisocytosis, macrocytosis (D-1)

Compared to pre-administration values, no changes in RBC indices were found in M25, G25 and M50 groups, while in G50 a significant decrease in mean cell haemoglobin concentration (MCHC) was found. In the high and moderate dose groups (M100, G100, M75, G75), the significant decrease in MCHC was accompanied by a significant increase in mean cell volume (MCV) and RBC distribution width (RDW-SD; Table 4). Representative pictures of the RBC shape alterations found in the high dose group 2 h after treatment administrations are shown in Fig. 1.

In line with these results, Pearson’s correlation analyses showed that the moles of glycerol and propylene glycol administered daily were directly correlated with RDW-SD (r = 0.539; *p* < 0.001) and MCV (r = 0.495; p < 0.001) and indirectly correlated with MCHC (r = 0.582; p < 0.001; Table 5). It should be pointed out that in all the groups but M100 these alterations were not observed in blood samples collected on the D 3 before glucogenic mixture administration, i.e., after 12 h from the last administration (Table 2).

**Table 5:** Pearson correlation coefficients between the moles of glucogenic compounds administered and RBC indices

	Moles of glycerol + propylene glycol	MCV (fL)	MCH (pg)	MCHC (g/L)
MCV (fL)	0.495 (<0.001)			
MCH (pg)	0.092 (0.573)	0.671 (<0.001)		
MCHC (g/L)	- 0.582 (<0.001)	- 0.675 (<0.001)	0.089 (0.586)	
RDW-SD (fL)	0.539 (<0.001)	0.846 (<0.001)	0.327 (<0.05)	- 0.809 (<0.001)

Corresponding *P*-values are shown within parenthesis

**Fig. 2:**
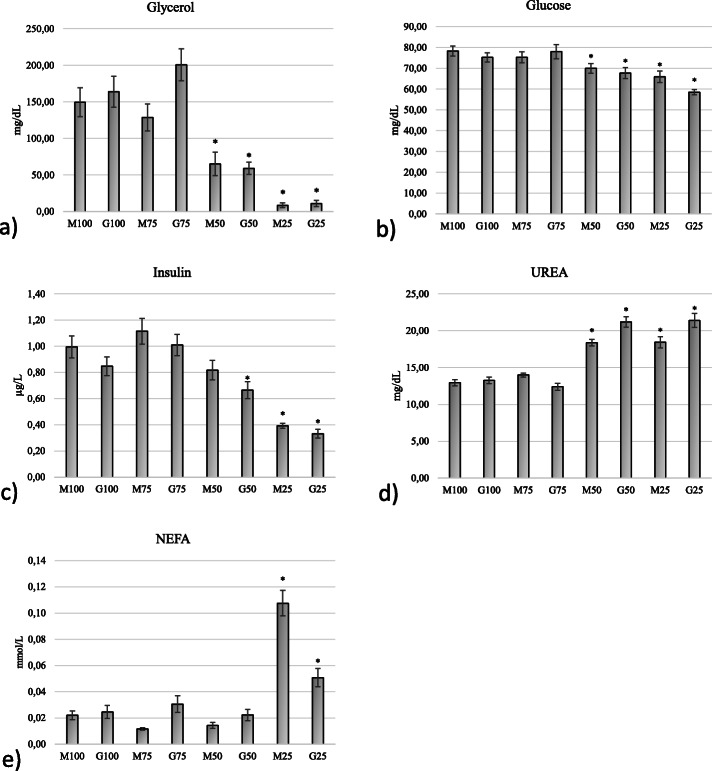
Mean circulating concentrations of glycerol, glucose, insulin, NEFA, and urea on day 3 of phase 1. Asterisks indicate significant differences between M100 group and the other groups (P < 0.05)

It should be pointed out that in all the groups but M100 these alterations were not observed in blood samples collected on the D 3 before glucogenic mixture administration, i.e., after 12 h from the previous administration (Table 1). To identify the formulations and doses capable to induce a positive change in ewe’s metabolic status we assessed their effects against those induced by M100. The latter dose has been indeed already evaluated for its effects on sheep metabolism and reproductive performance [8, 16, 18]. Obtained results shows that the high and moderate dose groups (G100, M75 and G75) showed no differences in mean glycerol plasma concentrations compared to the M100 one (Fig. 2a). Consequently, no differences were found between those groups and M100 in the circulating concentrations of glucose, insulin, urea and NEFA (Fig. 2b,c,d,e). Pearson’s correlation analyses showed indeed that the moles of glycerol and propylene glycol administered daily showed a fair direct correlation with glycerol, glucose, insulin and NEFA circulating concentration (r = 0.592; *p* < 0.001) and a fair indirect correlation with urea circulating concentration (r = 0.574; p < 0.001; Table 2).

In line with this results, the G50 and low dose groups (M25, G25) showed lower mean glycerol concentrations compared to M100, and consequently differed from M100 in the mean concentrations of glucose, insulin, and urea (Fig. 2b,d). It is noteworthy that M50, while differing from M100 in glycerol, glucose and urea mean concentrations, showed no difference in mean insulin concentrations (Fig. 2c). NEFA mean concentrations were higher than those found in M100 only in the low dose groups. Supplementary Figure 1 shows the concentrations-time data of the analysed metabolites and hormones.

Taken together, results show that the formulations M75 and G75 are able to induce metabolic changes comparable to those induced by M100. Hence, the dose of the glucogenic formulation can be lowered from 23 to 18% of diet on DM basis, without apparently inducing any significant change in ewe’s metabolic response. However, with these moderate doses, plasma osmolality increased significantly, and RBC indices showed significant variations. Between the medium dose group, M50 showed lower glucose and higher urea mean concentrations than M100. However, insulin and NEFA mean concentrations did not differ from those found in the M100 group. Moreover, the M50 group did not show any significant variations in plasma osmolality and RBC indices thus suggesting that glycerol osmotic effects were less marked than in the high and moderate dose groups.

Therefore, the glucogenic doses selected to determine the promotion of ovarian function (phase 2 of the present study) turned out to be M75, G75 and M50.

### Phase 2

As in the previous phase, body weight did not change after the administration of the feeding treatments and no differences were observed between groups (M75 42.3 ± 1.7, G75 42.4 ± 1.8, M50 44.4 ± 1.8, GON 42.1 ± 3.5; *P* > 0.05).

The number of follicles ranged between 1 to 3 mm in diameter, ovarian volume (mL), vascularity index, flow index and their relationship (Table 6) did not show any differences between groups. The number of large follicles (4 to 7 mm in diameter), however, was higher in the M75 group compared to the GON group (P = < 0.05; Table 6), while no differences were found between the other groups.

**Table 6:** Number of follicles, vascularity and flow index, their relationship and ovulation rate

Groups	Days	1–3 mm Follicles	4–7 mm Follicles	VOML	VIO	FIO	VFIO	Ovulation Rate (day 15)
M75	1	2.7 ± 0.7	3.0 ± 0.6	1.4 ± 0.1	2.5 ± 4.8	30.3 ± 3.2	8.2 ± 2.4	1.4 ± 0.2
	2	4.0 ± 0.0	2.7 ± 0.9	1.4 ± 0.2	26.4 ± 6.1	30.2 ± 3.5	9.0 ± 2.5	
	3	4.3 ± 0.3	2.3 ± 0.3	1.0 ± 0.1	31.9 ± 5.7	26.8 ± 2.1	9.3 ± 1.9	
	4	4.8 ± 0.7	2.6 ± 0.5 ^A, B^	0.9 ± 0.2	24.4 ± 10.3	27.5 ± 5.6	9.2 ± 5.1	
G75	1	4.0 ± 0.8	2.5 ± 0.5	1.7 ± 0.6	19.0 ± 3.9	28.3 ± 5.9	6.5 ± 2.5	1.6 ± 0.2
	2	4.8 ± 0.9	2.5 ± 0.6	2.4 ± 0.6	29.5 ± 5.0	31.6 ± 1.6	9.4 ± 1.0	
	3	4.8 ± 0.8	3.0 ± 0.4	1.3 ± 0.2	31.0 ± 8.5	89.6 ± 6.3	10.5 ± 3.5	
	4	3.5 ± 0.9	4.3 ± 0.5 ^A^	1.0 ± 0.1	8.5 ± 2.3	19.5 ± 1.1	1.7 ± 0.5	
M50	1	2.6 ± 0.4	2.0 ± 0.7	1.1 ± 0.2	2.1 ± 5.7	22.5 ± 3.8	6.2 ± 2.4	2 ± 0.2
	2	3.8 ± 0.6	1.3 ± 0.6	1.0 ± 0.2	4.0 ± 9.3	27.6 ± 3.3	12.9 ± 4.8	
	3	3.6 ± 0.7	2.0 ± 0.5	0.9 ± 0.2	2.2 ± 5.6	23.5 ± 3.0	6.3 ± 1.9	
	4	3.6 ± 0.9	2.8 ± 1.0 ^A, B^	1.2 ± 0.3	10.7 ± 1.3	19.8 ± 1.0	2.2 ± 0.3	
GON	1	2.5 ± 1.5	1.5 ± 0.5	1.4 ± 0.3	39.8 ± 6.6	37.6 ± 3.9	14.9 ± 3.3	1.4 ± 0.2
	2	4.0 ± 1.0	1.0 ± 0.6	1.4 ± 0.3	28.9 ± 6.3	29.4 ± 3.7	9.6 ± 3.2	
	3	5.3 ± 0.6	2.0 ± 0.4	1.4 ± 0.4	30.1 ± 9.6	32.2 ± 4.2	10.5 ± 4.7	
	4	5.0 ± 0.7	2.2 ± 0.4 ^B^	0.8 ± 0.2	28.8 ± 12.4	29.8 ± 6.6	9.7 ± 4.2	

Different letters indicate significant differences in the mean values of follicles recorded from D 1 to D 4 between groups ANOVA *p* < 0.05

VOML: 3D colour ovary perfusion; VI: vascular index; FI: flow index; VFI: blood vessels and blood flow index (VFI)

On the other hand, no differences were found between groups in oestradiol concentrations on Day 5 of experimental period (M50 16.2 ± 1.9 pg/mL; M75 12.6 ± 1.9 pg/mL; G75 14.8 ± 1.9 pg/mL; eCG 12.1 ± 2.1 pg/mL; *P* = 0.414). Finally, ovulation rate in M75 was equal to eCG (1.4 ± 0.2 for both) and even numerically higher in G75 and M50 (1.6 ± 0.2 and 2.0 ± 0.2, respectively), although differences were not statistically significant.

## Discussion

The results of the present study showed, for the first time, that the administration of glycerol-based glucogenic formulations causes significant changes in RBC indices of ewes with a dose-related effect. When the glucogenic formulations were administered at a percentage of inclusion on DM basis ranging from 18.2 to 27.4%, these changes indicated an increase in RBC volume (MCV), which was associated with a variation in the size of the erythrocytes (increased RDW-SD) and with a dilution in the amount of haemoglobin per volume unit (decreased MCHC). In the high and moderate dose groups the RBC shape alterations were found 2 h after the administration of glucogenic formulation administration when glycerol concentrations in the bloodstream were higher than ≈ 200 mg/dL. This rise was also associated with a significant increase in plasma osmolality, as already reported in lactating ewes when the glycogenic formulation was given at 23% of inclusion on DM basis [21]. When the glycerol formulations were administered at lower doses (G50, 15.9% DM) a significant decrease in MCHC was the sole alteration observed. Below such value (≤ 12.9% DM; M50, M25 and G25), no significant alterations in RBC indices were found.

Glycerol is lipid soluble so it can diffuse by simple diffusion directly through the RBC membrane following a concentration gradient [32]. It is known that glycerol enters human RBCs by two pathways: facilitated diffusion through aquaporins, and simple diffusion [40, 41]. By contrast, glycerol is transported in sheep RBC via simple diffusion only [41, 42], and hence it has a lower permeability coefficient compared to humans [43]. In humans, the effects of glycerol permeation on RBC functionality have been described in in vitro studies focusing on RBC cryopreservation, where glycerol is used as penetrating cryoprotectant [44]. It has been reported that glycerol permeation in the RBC membrane resulted in shape alterations (increased MCV and RDW-SD) which appeared to be closely related to membrane alterations, increased permeability to ions and increased osmotic fragility [45]. These results are confirmed by another study in which the glycerol- dependent shape alterations (increased MCV and decreased MCHC) were also associated with an electrolyte shift [34]. Glycerol is indeed a polar molecule which is known to alter the ionic strength and the dielectric constant of aqueous solutions [46]. In sheep, incubation of RBCs in glycerol media causes haemolysis due to a gradual increase of the intracellular glycerol concentration [47]. We can thus speculate that the RBC shape alterations observed in the high dose groups of this investigation may be caused by the glycerol permeating cell membrane following a concentration gradient [32] generated by the significant rise in glycerol circulating concentrations consequent to the dietary administration. Shape alterations of sheep RBCs reported in this trial are similar to those described in human glycerolized RBCs [34, 45], and might thus be associated with erythrocyte impaired functionality. In addition, when the average size of RBC’s increases, they can fail to pass the capillaries in the microcirculation and be removed from the circulation [48]. All these changes might thus contribute to enhanced eryptosis. Further studies are needed to quantify the permeation of glycerol in the RBCs in vivo and to extend our knowledge on its osmotic effects. In the present study, in all groups but M100 the RBC shape alterations were not observed in blood samples collected 12 h after the glucogenic mixture administration. Nevertheless, reported results suggest the need to lowering the dose of the glycerol administered both as replacer of corn grain and as feed supplement, especially in long-lasting protocols.

By lowering the dose, however, the efficacy should be guaranteed. To verify whether the tested formulations were able to create a metabolic milieu suitable for the final follicular growth and thus for the conception period in the ewe, as described in previous studies [20], we compared the circulating concentrations of glycerol, glucose, insulin, urea and NEFA against those found in the M100 dose. This dose was taken from previous studies, being already evaluated for its effects on sheep metabolism and reproductive performance [8, 16, 18]. In the low dose groups, showing no alterations in plasma osmolality and RBC indices, the metabolic milieu was markedly different compared to that found in M100 (lower glycerol, glucose and insulin circulating concentrations, higher NEFA and urea circulating concentrations). In the high and moderate dose groups (G100, G75 and M75) the metabolic milieu elicited did not differ significantly from M100. However, plasma osmolality and RBC indices showed significant alterations compared to pre-administration values.

The medium dose groups, despite providing the same amount of energy (0.6 Mcal/d of NE_L_), differed both for the metabolic response and for the RBC indices alterations observed. When this amount of energy was supplied by an association of glycerol and propylene glycol (M50), the metabolic milieu elicited was more similar to that found in M100 and no alterations in plasma osmolarity and RBC indices were observed. If the same amount of energy was supplied by glycerol alone (G50), the total dose of glycerol administered daily increased from 70 to 110 mL. This higher dose was associated with RBC indices alterations (increased MCHC). In addition, insulin concentrations were lower than those found in M100. The association of glycerol with propylene glycol seems thus a better option than glycerol alone. The hyperinsulinemic effect of propylene glycol is related to its glucogenic effect consequent to the increased absorption of propanol, propionate, and propanal originating from ruminal metabolism of propylene glycol [49]. This effect could be related to insulin resistance consequent to increased circulating levels of propylene glycol and propanol or to a decrease in the ratio of ketogenic to glucogenic metabolites in plasma [49].

However, if administered at high doses propylene glycol fermentation may produce sulphur-containing gases [50] and provoke negative effects, including ataxia, salivation, hyperventilation and depression [51]. At low doses, however, it allows reducing the amount of glycerol administered, and hence its osmotic effects, while acting synergistically in rising insulin concentrations.

The rise in glucose and insulin directly promotes follicular growth and development, mainly by altering FSH-induced effects on the synthesis of oestradiol by the granulosa cells, in accordance with the availability of glucose [52]. The follicle has indeed a functional insulin-glucose-IGF-1 system which is affected by short-term nutritional treatments, and components of this metabolic system are nutritionally regulated in the follicle [53]. Moreover, elevated circulating concentrations of NEFA, which are indicative of negative energy balance, affect follicular growth and fertility by acting directly on the different ovarian cells, including the oocyte within the growing follicle, and may also affect the development of the fertilized oocyte, morula and early blastocyst [52]. Therefore, diets or metabolic states that favour high concentrations of NEFAs should be avoided during the cycle of conception and the early post-conception period [52]. In the same way, high levels of urea in blood have been associated with lower fertility due to a changed uterine environment and poor embryo viability [54–56].

The administration of M50 was associated with an increase in the mean circulating concentrations of glucose, although less marked than that found in M100, and insulin and with a decrease in NEFA circulating concentrations. Taken together, these results suggest that the M50 may represent a safe glucogenic formulation in ewes, being able to elicit comparable metabolic conditions as found at higher doses, but without altering RBC indices and plasma osmolality. The effects at ovary levels were thus compared with those of moderate doses of glucogenic formulation (M75 and G75) and with those provoked by eCG, having an FSH/LH-like effect. The use of gonadotropins is routinely incorporated into synchronization systems used in ewes to induce ovulation. The most commonly used product is eCG because, besides inducing ovulation, such hormone reduces variability in the onset of oestrus among treated animals and increases ovulation rate as a consequence of a greater follicular development [57]. In the present study, all glucogenic formulations were able to elicit an ovulatory response which did not differ significantly from what found in eCG treated ewes. It is remarkable that the high doses (G75 and M75) did not overcome the medium one (M50), which in contrast showed a numerically higher ovulation rate than the other treatments (n.s).

## Conclusions

Glycerol is widely used as a feed supplement in ruminant nutrition. However, the results of the present study showed an alteration of RBC indices, and possibly of their functions, as a side effects of glycerol administration at moderate and high doses. Therefore, protocols foreseeing the administration of glycerol should be tested for their effects on RBC indices and functions. In general terms, medium doses of glucogenic mixtures should be preferred for flushing dairy ewes, as they proved to be effective at metabolic level without causing alterations in RBC indexes and possibly of their functionality.

## Methods

The experiments were carried out at Bonassai research station of Agris Sardegna, located in north-western Sardinia, Italy (40 °N, 8 °E, 32 m a.s.l.). This study was divided into two experimental phases. Phase 1 aimed at determining the lowest doses of glycerol as a titration feeding trial able to modify ewe’s metabolic status, as previously described using a high dose [20], without impacting on RBC indices. Glycerol was administered both alone and in combination with propylene glycol, as previously described [10, 19, 20]. Phase 2 aimed at assessing the effect of the administration of the glucogenic doses selected in Phase 1 on ovarian follicular population assessed by three-dimensional ovarian ultrasound scanning and oestradiol production during an induced follicular phase. The ewes enrolled in phase 1 and 2 were obtained from the experimental flock reared at the Agris research station. After the study, the ewes were released and returned to the experimental flock. Based on the research objective, the minimum sample size was estimated in order to obtain significantly reliable data on differences in glucose plasma levels. This estimation derived from the use of common methods for statistical evaluation, allowing to test the quality of different glucogenic-based nutritional treatments using the minimum number of animals. For this purpose, a power analysis was carried out using the statistical software G * Power 3.1.9.2, setting the α error at 0.05 and the statistical power at 0.80. The analysis showed that, with a total of 5 animals per group, there is an 81% chance of correctly rejecting the null hypothesis of absence of differences between groups.

### Phase 1

#### Animals and treatments

The experiment was run during March–April 2019, within the anoestrus season described for this breed at this latitude. Twenty adult (3–7 years) and multiparous non-lactating Sarda dairy ewes were used. Glycerol and propylene glycol had a purity grade of 99.5–100% and complied with EU Reg. 231/2012 for food additives (E422 and E1520 for glycerol and propylene glycol, respectively; Farmalabor srl, Assago, Milano, Italy). Glycerol was administered alone (G groups: 90% glycerol and 10% water; % v/v) or in combination with propylene glycol (M groups: 70% glycerol, 20% propylene glycol, 10% water; % v/v). Treatments were formulated in order to give 100, 75, 50 and 25% of the amount of energy supplied in previous experiments [10, 20, 21]. Table 7 summarizes ingredient and nutrient composition of the dietary formulation along with estimated energy supplied to each group. A two-period design was used to allow comparisons between the eight formulations tested (M100, G100, M75, G75, M50, G50, M25, G25), with 4 days of treatment (period 1, groups M100, G100, M25, G25, *n* = 5 per group), followed by 1 month of wash out and 4 days of treatment (period 2, groups M75, G75, M50, G50, n = 5 per group). Each ewe was randomly assigned to 2 treatment groups, one per period. Groups were homogeneous for body weight (BW; Table 7), measured before the morning meal by means of a digital scale for livestock.

**Table 7:** Ingredient and nutrient composition of the dietary formulation along with estimated energy supplied

	High doses		Moderate doses		Medium doses		Low doses	
Group	M100	G100	M75	G75	M50	G50	M25	G25
BW	53.3 ± 2.0	55.2 ± 2.3	51.5 ± 1.7	50.7 ± 1.3	49.0 ± 2.0	49.2 ± 1.8	49.0 ± 2.0	49.7 ± 2.3
Formulation	Glycerol, Propylene glycol, Water	Glycerol, Water	Glycerol, Propylene glycol, Water	Glycerol, Water	Glycerol, Propylene glycol, Water	Glycerol, Water	Glycerol, Propylene glycol, Water	Glycerol, Water
Energy %	100%	100%	75%	75%	50%	50%	25%	25%
Energy (NE_L_ Mcal/d)	1.2	1.2	0.9	0.9	0.6	0.6	0.3	0.3
Glycerol (mL)	140	220	105	169	70	110	35	55
Propylene Glycol (mL)	40		30		20		10	
Water (mL)	20	22	15	17	10	11	5	5
Total (mL)	200	242	150	186	100	121	50	60
% DM	22.9	27.4	18.2	22.5	12.9	15.9	6.9	8.6

Glucogenic formulations were administered b.i.d. for 4 days (D 1–4 of the experimental periods) at 08:00 in the morning and 19:00 in the evening, using a drench gun. This daily dosing schedule was set as close as possible to that adopted in previous experiments by our laboratories (twice daily, every 12 h e.g.) [10].

During each period, before starting the glucogenic treatment (D 0 of the experimental period), blood samples were collected at fasting (08:00). In addition, on D 3 of the experimental period, consecutive blood samples were collected at 8 time points, starting at fasting immediately before the morning administration of glucogenic mixture (08:00, 08:30, 09:00, 09:30, 10:00, 11:00, 12:00, 18:00). From.

**Fig. 3:**
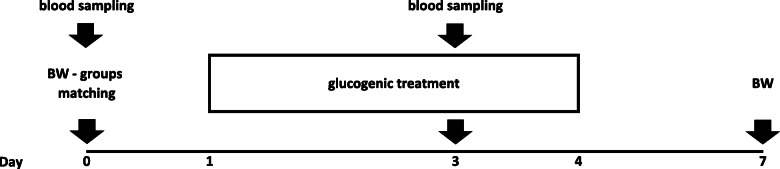
Phase 1 experimental protocol. Figure shows exact timing at which treatments, samplings and measurements were performed

D 0 to D 7, i.e. throughout the treatment periods, the sub-groups were kept indoors in separate pens. Indoor daily feeding consisted of 200 g/head of a pelleted concentrate individually fed at 08:00, plus c.a. 1500 g/head of ryegrass hay. On blood sampling day, the concentrate was fed immediately after the first bleeding. The pelleted concentrate was completely consumed by the animals. Water and mineral blocks were available ad libitum. During the wash out period, the ewes of both experimental subgroups were fed the same diet as above. Figure 3 shows the exact timing at which treatments, samplings and measurements were performed.

#### Feedstuff composition

The compound feed was a commercial pelleted formulation based on cereals and soybean meal, with a net energy content (NE_L_) of 1.43 Mcal/kg, 17% Crude protein (CP) and 32% starch, on DM basis. The home-grown hay was a late-cut Italian ryegrass with 6.5% CP and 75% NDF and a NE_L_ content of 0.86 Mcal/kg on DM basis. The hay was chopped before feeding.

#### Blood samplings and plasma osmolality determination

On D 0, in both periods, plasma concentrations of glycerol, glucose, insulin, NEFA, urea, plasma osmolality and, in whole blood, RBC indices were determined from samples drawn from jugular vein before morning feeding at 08:00. On D 3, plasma concentrations of glycerol, glucose, insulin, NEFA, urea, and plasma osmolality were also determined from eight consecutive samples, while RBC indices from two samples (at 08:00 and at 10:00). Repeated sampling on D 3 were collected after jugular cannulation. At each sampling, from each ewe, two blood samples were collected: one using 2-mL vacuum collection tubes with glycolytic inhibitor (5.0 mg sodium fluoride, 4.0 mg pot. ox. - Vacutainer Systems Europe; Becton Dickinson, MeylanCedex, France) for glucose assay; one using 2.0 mL vacuum collection whole blood tube with spray-coated K_2_EDTA (Vacutainer Systems Europe; Becton Dickinson, MeylanCedex, France) for other metabolites, insulin and RBC indices determination. Immediately after recovery, samples were cooled to 4 °C. RBC indices were determined within 2 h of blood collection. The other blood samples were centrifuged at 1500 g for 15 min at 4 °C degrees. Individual plasma was removed and stored in vial at − 20 °C until assayed.

Plasma osmolality (Osm/kg) was measured using a freezing point osmometer (Osmomat 030, Gonotec, Berlin, Germany).

#### Complete blood count (CBC)

Haematological testing was integral to the interpretation of results to establish the safe amount of glycerol following the titration trial. Particular care was taken to prevent analytical biases responsible to affect blood sample diagnostic potentials. In light of the recommendations of the International Council for Standardization in Hematology (ICSH) the use of K_2_EDTA (ethylene diamine tetracetic acid) as anticoagulant was preferred, due to the less pronounced osmotic effect on blood cells than that exerted by K_3_EDTA [58, 59]. O The same skilled veterinary surgeon collected blood samples in vacuum tubes until filled to the correct volume and gently mixed 5 to 10 times to allow complete interaction with the anticoagulants and to prevent clotting [58], before storage in the upright position and cooling to 4 °C. According to the recommendations to preserve diagnostic samples in the preanalytical phase (ICSH, 1993), all the samples were transported refrigerated to the laboratory where individual haematological profiles were assessed within 2 h of blood sampling.

In brief, 15 μL of each whole blood sample were needed for the determination of haematological parameters through capillary analysis based on tri-angle scattering and chemical dying read through flow cytometry technology. The following parameters were determined using an automatic cell counter instrument (Hematology analyzer Alcyon Mindray BC-5000, Shenzhen, China): white blood cell count (WBC), red blood cell (RBC), hemoglobin (HGB), hematocrit (HCT), mean corpuscular volume (MCV), mean corpuscular hemoglobin (MCH), mean corpuscular hemoglobin concentration (MCHC), red blood cell distribution width (RDW-SD), platelet (PLT), neutrophil granulocytes (Neu), lymphocytes (Lym), monocytes (Mon), eosinophil granulocytes (Eos), basophil granulocytes (Bas), mean platelet volume (MPV), platelet distribution width (PDV) and plateletcrit (PCT). Both absolute and relative values of leukocytes were analyzed for each sample. For the purposes of the present study, only the following RBC indices [60] were considered: 1) MCV: defines the size of the red blood cells and is expressed as femtoliters (10^− 15^; fl); 2) MCH: quantifies the amount of haemoglobin per red blood cell and is expressed in picograms (pg); 3) MCHC: indicates the amount of haemoglobin per unit volume. In contrast to MCH, MCHC correlates the haemoglobin content with the volume of the cell and it is expressed as g/L of RBCs; 4) RDW-SD: represents the coefficient of variation of the RBC volume distribution (size) and is expressed as femtoliters. It is a good indicator of the degree of anisocytosis.

In addition, from each blood sample processed for CBC, whole blood smears were carried out for microscopic examination of blood cell morphology. In particular, microscopic examination (200X; Olympus BX41, Olympus Italia Srl, Segrate, Milano, Italy) of whole blood smear aimed to support the automatic reading obtained at haematology analyser, by excluding the presence of accidental platelets clumping or RBC rouleaux formation and therefore to consider the samples as diagnostic for the purpose of the investigation.

#### Metabolites

Plasma samples were measured in duplicate. Glycerol concentration was measured in a single assay by colorimetric method using a commercial Free Glycerol Assay Kit (Cell Biolabs, Inc., USA), with glycerol standards in the concentration range of 0 μM–400 μM. The kit measures free, endogenous glycerol by a coupled enzymatic reaction system. The glycerol is phosphorylated and oxidized, producing hydrogen peroxide which reacts with the kit’s Colorimetric Probe (absorbance maxima of 570 nm). The analytical detection limit was 5 μM.

Glucose, NEFA, and urea were measured using commercial kit and BS-200 Mindray clinical chemistry analyzer. We used Serum I Normal (Wako) and Serum II Abnormal (Wako) as multi control for each measured parameter. Glucose concentrations were determined in a single assay by liquid enzymatic colorimetric method (GOD-POD) (Real Time kit) with a glucose standard of 100 mg/dL for calibration. Intra-assay CV values were 1.1%. NEFA and urea concentrations were measured in multiple assays by enzymatic endpoint method (Diagnostic Systems kit), with a NEFA standard of 1 mmol/L and a urea standard of 50 mg/dL for calibration. NEFA intra-assay and interassay CV values were 1.07 and 0.98%, respectively. UREA intra-assay and interassay CV values were 1.7 and 1.6%, respectively.

#### Insulin

ELISA assays were performed using the Personal Lab Adaltis (Adaltissrl, Rome, Italy), which is a tool that performs automated ELISA protocols. Insulin concentration was measured in duplicate using a commercial Ovine Insulin ELISA Kit (Mercodia developing diagnostics, Germany) which is a solid-phase ELISA based on the direct sandwich technique. The kit is calibrated against an in-house reference preparation of ovine insulin, and it has been previously used for insulin determination in ovine plasma [61, 62]. The mean ovine insulin concentrations of the six reference solutions were 0, 0.05, 0.15, 0.5, 1.5, and 3 mg/L. The recovery on addition was 94–114% (mean 103%). The analytical sensitivity was 0.025 mg/L and the intra-assay and interassay CV values were < 7%.

### Phase 2

#### Animals and treatments

The experiment was run during August–September 2019, within the natural breeding season described for this breed at this latitude. Twenty adults (2–7 years) non-lactating Sarda dairy ewes were used. The glucogenic formulations to be tested (G75, M75, M50) were chosen on the basis of the results of Phase 1. Control ewes (GON group) received 150 mL of water twice daily simultaneously to treatment administration. Water was administered using a drench gun. The four experimental groups were homogeneous for body weight (BW, mean Kg ± SE; GON 43.6 ± 3.7, *n* = 5; G75 43.8 ± 1.8, n = 5; M50 44.2 ± 1.9, n = 5; M75 43.4 ± 1.2, n = 5; *P* = 0.996).

**Fig. 4:**
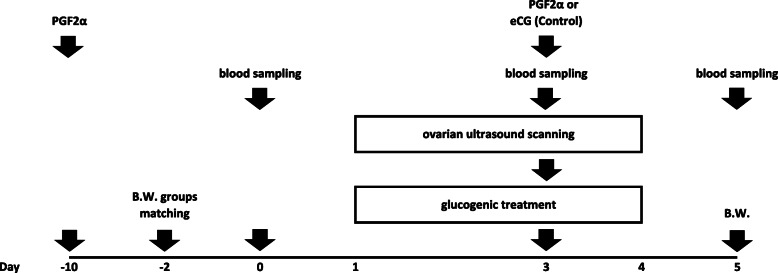
Phase 2 experimental protocol. Figure shows exact timing at which treatments, samplings and measurements were performed

Glucogenic treatments were administered following an oestrus synchronization protocol. In brief (Fig. 4), synchronization was induced in all the animals by i.m. injection of PGF2α analogue (PGF Veyx 0,250 mg/mL, Veyx Pharma GMBH, Schwarzenborn, Germany), administered twice, at 13 d interval (first injection at day − 10, second at day 3). Simultaneously to the second PGF2α injection (D 3, 08:00) control ewes (GON group) were injected intramuscularly with 200 IU of eCG (Folligon, MSD Animal Health Srl, Segrate, Italy).

From D 0 to D 4, i.e. throughout the treatment periods, the groups were kept indoors in separate pens. Indoor daily feeding consisted of 200 g/head of a commercial pelleted feed and 150 g/head of maize grain divided in two equal meals (08:00 and 15:00) and individually fed in the milking parlour and 1500 g/head of hay fed in the afternoon. On the blood sampling day, the morning meal based on compound feeds was administered immediately after the first blood sampling. Water and mineral blocks were available ad libitum. Concentrates were completely consumed by the animals.

#### Feedstuff composition

Compound feeds and hay administered in the phase 2 were the same as those of phase 1. The whole maize grain had CP content of 7.80%, starch level of 69.9% and a NE_L_ of 2.00 Mcal/kg on DM basis.

#### Determination of oestradiol plasma concentration

On day 5 of the experimental period (48 h after the second PGF2α administration), blood samples were drawn from jugular vein at fasting at 08:00. Blood samples were collected using 10-mL vacuum collection tubes (BD Vacutainer® Rapid Serum Tube - Vacutainer Systems Europe; Becton Dickinson, MeylanCedex, France) for oestradiol assay. Immediately after recovery, blood samples were cooled at 4 °C and centrifuged at 1500 g for 15 min. Serum was removed and stored at − 20 °C until assayed.

Samples were measured in duplicate. Concentrations of oestradiol were measured after sample extraction by using a highly sensitive commercial enzimoimmunoassay kit for quantitative determination of estradiol-17β (Demeditec Diagnostics GmbH, Kiel-Wellsee, Germany*).* Sensitivity of the assay was 1.4 pg/mL and intra-assay variation coefficient was 5.7%.

#### Ovarian 3D ultrasound scanning

From day 1 to day 4, ovarian follicular population was studied daily using trans-vaginal 3D ultrasonography. Ultrasonographic examinations were carried out using a Mylab-alpha (Esaote, Italia) fitted to an endocavitary transducer (8–11 MHz). Each ovarian follicular population was scanned using both conventional 2D-US, 3D-US and 3D Power Doppler Ultrasound (3D PD-US) image acquisition methods by an experienced sonographer. 3D power Doppler were performed to evaluate the ovary perfusion.

The images were recorded as digital files for later examination and the ovarian follicular population vascular index (VI), flow index (FI), blood vessels and blood flow index (VFI) by three-dimensional Doppler histogram were calculated.

Thereafter, on day 14 ovulation rates were determined by counting the corpora lutea present in each ovary by transrectal ultrasonography with a real-time B-mode scanner (Aloka102 SSD 500; Aloka Co., Tokyo, Japan) fitted with a 7.5 MHz linear-array probe.

### Statistical analyses

Results are expressed as mean values (mean ± SE) or median values (median and range) and the differences were considered to be statistically significant at *P* < 0.05.

Differences in ewe’s body weight at the beginning and at the end of the glucogenic-treatment period in phase 1 and 2 were analysed by a mono-factorial GLM.

The distribution of variables at Day 0 was assessed by the Kolmogorov–Smirnov test.

In phase 1, longitudinal data of plasma glycerol, glucose, insulin, NEFA and urea in the consecutive samples collected on day 3 (during treatment period) were analysed by a GLM UNIANOVA model in SPSS (IBM Corp. Released 2016. IBM SPSS Statistics for Windows, Version 24.0. Armonk, NY: IBM Corp) with treatment, sampling hour and their first-order interactions as fixed effects. As post-hoc test, for plasma glycerol, glucose, insulin, NEFA and urea, a one-tailed Dunnett’s test was used to highlight negative or positive differences, in respect of M100 group, as appropriate.

In addition, mean circulating concentration of plasma glycerol, glucose, insulin, NEFA and urea on day 3 (during treatment period) were analysed by a GLM UNIANOVA model in SPSS with treatment as fixed effects. As post-hoc test, a one-tailed Dunnett’s test was used to highlight negative or positive differences, respect to M100 group, as appropriate.

A SPSS GLM UNIANOVA model was also used to highlight changes among groups on plasma osmolality, RBC indices and circulating concentrations of analysed metabolites and hormones between day 0 and day 3.

Longitudinal data of plasma osmolality in the consecutive samples collected on day 3 (during treatment period) were analysed by a GLM UNIANOVA model in SPSS with treatment, sampling hour and their first-order interactions as fixed effects. A post-hoc test Tukey’s test was used to highlight differences between and among groups.

A SPSS GLM UNIANOVA model was also used to evaluate within groups the effect of nutritional treatments on RBC indices before and 2 h after treatment administration on D 3.

Finally, the relationship between treatments administrated, concentration of metabolites and hormones and the relationship between treatments and RBC indices at two hours post treatment, were evaluated by Pearson’s correlation analysis. The strength of the correlation was considered poor for r values ranging from ±0.1 to ±0.3, fair for r values ranging from ±0.3 to ±0.5, moderately strong for r values ranging from ±0.6 to ±0.8 and very strong for r values ranging from ±0.8 to ±1 [63].

In phase 2, longitudinal data on follicular population, ovarian vascularity and flow index and their relationship, as evaluated by ultrasound scanning, were analysed by a GLM UNIANOVA model in SPSS with treatment, day and their first-order interactions as fixed effects. As post-hoc test, Fisher LSD test was used to highlight differences between groups. A One-way ANOVA test was used to compare ovulation rate and oestradiol blood levels between the different experimental groups.

## Supplementary information

**Additional file 1: Figure 1.** – Concentrations-time data of the analysed metabolites and hormones on day 3 of phase 1. Asterisks indicate significant differences between M100 group and other groups (*P* < 0.05).

## Data Availability

The datasets used and analysed in the current study are available from the corresponding author on reasonable request.

## Abbreviations

RBC: red blood cell
eCG: equine chorionic gonadotrophin
DM: dry matter
MCV: Mean corpuscular volume
MCH: Mean corpuscular hemoglobin
MCHC: Mean corpuscular hemoglobin concentration
RDW-SD: red blood cell distribution width
NEFA: Non-esterified fatty acids
VOML: 3D colour ovary perfusion
VI: Vascular index
FI: Flow index
VFI: Blood vessels and blood flow index
BW: Body weight
NE_L_: Net energy content
CBC: Complete blood count
