# Supplementary figures and images for: Administration of glycerol-based formulations in sheep results in similar ovulation rate to eCG but red blood cell indices may be affected
Source: BMC Vet Res. 2020 Jun 22;16:207. doi: 10.1186/s12917-020-02418-z (PMC7310049; doi:10.1186/s12917-020-02418-z)

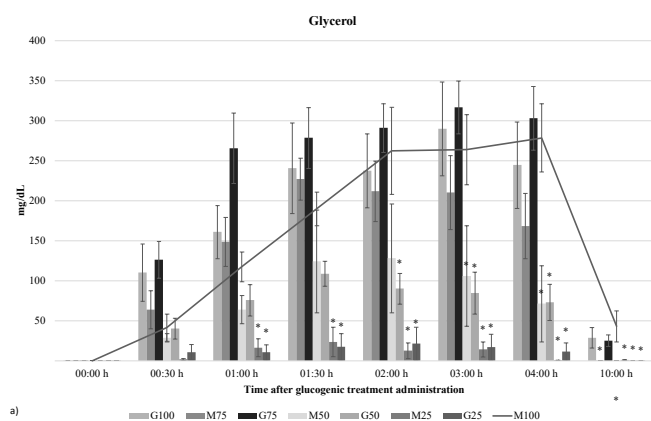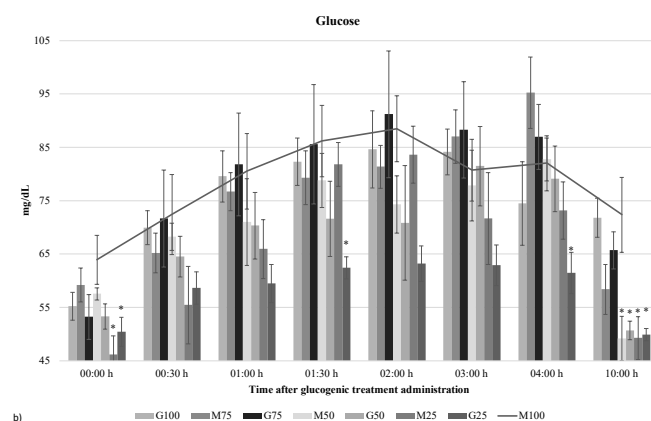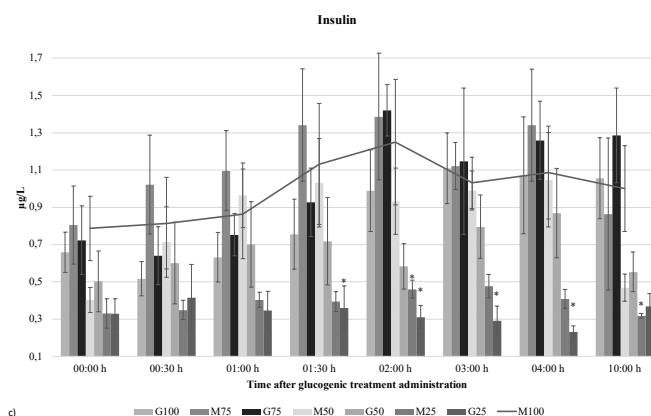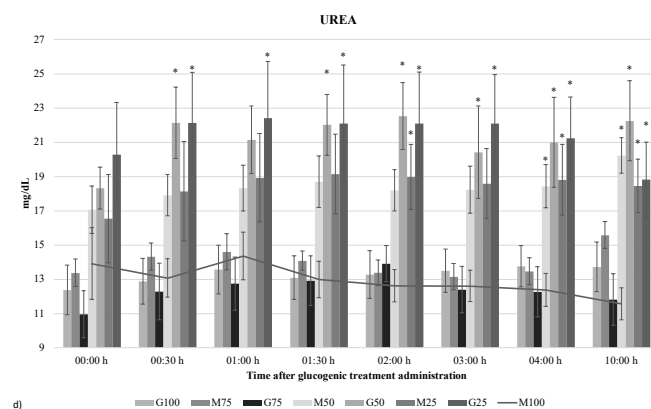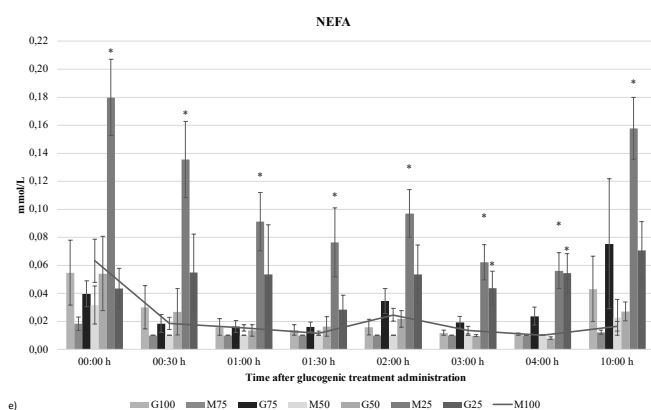

Supplement: Supplementary file 1 — Additional file 1: Figure 1. – Concentrations-time data of the analysed metabolites and hormones on day 3 of phase 1. Asterisks indicate significant differences between M100 group and other groups (P < 0.05). [file 12917_2020_2418_MOESM1_ESM.pdf]
